# Design and Properties of Ligand-Conjugated Guanine Oligonucleotides for Recovery of Mutated G-Quadruplexes

**DOI:** 10.3390/molecules23123228

**Published:** 2018-12-06

**Authors:** Shuntaro Takahashi, Boris Chelobanov, Ki Tae Kim, Byeang Hyean Kim, Dmitry Stetsenko, Naoki Sugimoto

**Affiliations:** 1 Frontier Institute for Biomolecular Engineering Research (FIBER), Konan University, 7-1-20 Minatojima-Minamimachi, Chuo-ku, Kobe 650-0047, Japan; shtakaha@konan-u.ac.jp; 2 Institute of Chemical Biology and Fundamental Medicine, Siberian Branch of the Russian Academy of Sciences, 8 Lavrentiev Ave., 630090 Novosibirsk, Russia; boris.p.chelobanov@gmail.com (B.C.); dmitry.stetsenko@ntlworld.com (D.S.); 3 Novosibirsk State University, 2 Pirogov Str., 630090 Novosibirsk, Russia; 4 Department of Chemistry, Division of Advanced Materials Science, Pohang University of Science and Technology (POSTECH), Pohang 37673, Korea; mcmurry@postech.ac.kr (K.T.K.); bhkim@postech.ac.kr (B.H.K.); 5 Graduate School of Frontiers of Innovative Research in Science and Technology (FIRST), Konan University, 7-1-20 Minatojima-Minamimachi, Chuo-ku, Kobe 650-0047, Japan

**Keywords:** G-quadruplex, ligand, replication, cancer

## Abstract

The formation of a guanine quadruplex DNA structure (G4) is known to repress the expression of certain cancer-related genes. Consequently, a mutated G4 sequence can affect quadruplex formation and induce cancer progression. In this study, we developed an oligonucleotide derivative consisting of a ligand-containing guanine tract that replaces the mutated G4 guanine tract at the promoter of the vascular endothelial growth factor (VEGF) gene. A ligand moiety consisting of three types of polyaromatic hydrocarbons, pyrene, anthracene, and perylene, was attached to either the 3′ or 5′ end of the guanine tract. Each of the ligand-conjugated guanine tracts, with the exception of anthracene derivatives, combined with other intact guanine tracts to form an intermolecular G4 on the mutated VEGF promoter. This intermolecular G4, exhibiting parallel topology and high thermal stability, enabled VEGF G4 formation to be recovered from the mutated sequence. Stability of the intramolecular G4 increased with the size of the conjugated ligand. However, suppression of intermolecular G4 replication was uniquely dependent on whether the ligand was attached to the 3′ or 5′ end of the guanine tract. These results indicate that binding to either the top or bottom guanine quartet affects unfolding kinetics due to polarization in DNA polymerase processivity. Our findings provide a novel strategy for recovering G4 formation in case of damage, and fine-tuning processes such as replication and transcription.

## 1. Introduction

The guanine quadruplex (G4) represents one of the non-canonical structures of nucleic acids. G4s are formed by four strands of DNA or RNA tracts containing a series of four guanines linked by Hoogsteen base-pairing. Stacking each guanine (G) quartet with a coordination metal cation (mainly K^+^ or Na^+^) stabilizes the G4 structure. Since G4 formation disrupts interactions in molecular machinery involved in replication and transcription, a G4 formed on genomic DNA is considered as a functional unit regulating gene expression [1,2]. Many G4 motifs are present particularly in the promoter regions of cancer-related genes [3]. Hence, a glitch in G4 formation may disrupt the expression of such genes, inducing cancer progression. G4 formation is affected by various environmental factors, such as K^+^ concentration and molecular crowding [4]. Furthermore, the formation of G4 is induced by the negatively super-coiled structure of double stranded DNA, and the structure of G4 is stabilized by certain G4-binding proteins [5]. Thus, disruption of the immediate intracellular environment plays a key role in cancer development. We previously reported that a decreasing intracellular K^+^ concentration is correlated with increasing cancer malignancy, by ways of G4 destabilization on cancer-related genes and the consequential upregulation of transcription [6]. Therefore, the development of G4 ligands that stabilize G4s in certain intracellular environments are expected to have considerable impact on cancer progression and provide an application in cancer treatment.

Enhanced oxidative status in cells is another intracellular environment associated with cancer progression. Oxidative stress caused by reactive oxygen species (ROS) triggers the oxidation of nucleobases in genomic DNA. As guanine exhibits the lowest oxidative-reduction potential among the DNA bases, guanine oxidation is the most prevalent [7]. Moreover, reports indicate that guanines in the G4 structure are relatively more sensitive to oxidation [8], making the G4 forming genomic sequences ‘hot spots’ of base oxidation. The major oxidation product of guanine is 8-Oxo-7,8-dihydroguanine (8-oxoG) [9,10]. The generation of 8-oxoG within a G4 forming sequence inhibits stable G4 formation and alters G4 topology [11]. Moreover, a replication error can cause a mutation substituting G with T at the position of 8-oxoG, thereby permanently disrupting G4 formation [12]. In cancer cells, 8-oxoG bases in G4-forming sequences within the promoter region of the vascular endothelial growth factor (*VEGF*) gene have been found to upregulate VEGF expression [9]. However, chemical approaches which may prove useful for recovering damaged G4 formations have not yet been explored. In a recent study, we developed a novel technique to recover the formation of damaged G4s, using pyrene-conjugated guanine tracts (PyG3) [11]. PyG3 forms (3 + 1) intermolecular G4 structures by replacing damaged guanine bases [13], significantly stabilizing intermolecular G4 formation by stacking of pyrene moieties to form G-quartets [11]. Therefore, the conjugation of suitable G4 ligands to short guanine-rich oligonucleotides can be considered as a general strategy for restoring G4 structures containing base lesions.

In the present study, guanine tracts conjugated with various polyaromatic hydrocarbons, including pyrene (Py), 9,10-bis(phenylethynyl)anthracene (BPEA), 1-phenylethynylpyrene (PEPy), and perylene (Per), were synthesized. Each ligand was covalently attached to either the 3′ or the 5′ end of the guanine tract by a phosphodiester group and a linker of a varying structure, length, and flexibility. These guanine tracts interacted with target mutated G4 at the VEGF promoter, containing a second guanine mutated to thymine due to a replication error for 8-oxoG, to form intermolecular G4 structures. Circular dichroism (CD) analysis indicated that stability as well as topology of the formed intermolecular G4s depended on the structure of the ligand moiety and the attachment position at the end of the guanine tract. We performed polymerase stop assays using template DNA with mutated VEGF G4 complexed with ligand conjugated guanine tracts. Results show that each guanine tract conjugated with a ligand at the 3′ end effectively stalls replication. These findings may prove useful for the design of effective treatments to restore G4 structures containing base lesions in cancer cells.

## 2. Results

### 2.1. Design of Ligand-Conjugated Guanine Tracts

**Figure 1:**
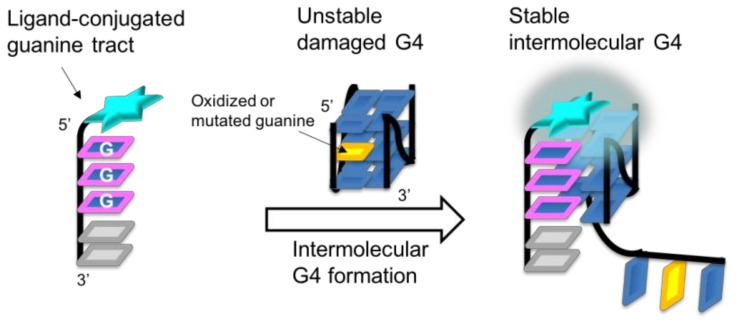
Schematic illustration of the recovery of mutated VEGF G4 by intermolecular G4 formation using a ligand-conjugated guanine tract.

**Figure 2:**
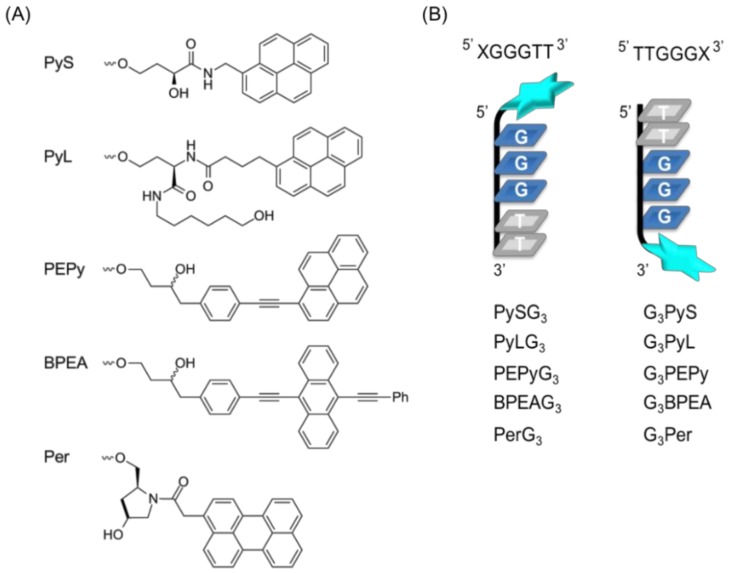
Structures of ligand-containing guanine oligonucleotides used in this study. (**A**) Chemical structures of the ligand-containing linkers. (**B**) Structures of ligand-conjugated guanine tracts. The attachment of a ligand-containing linker was either to the 3′ or 5′ OH group of the respective deoxyguanosine residue via the phosphodiester linkage.

A ligand-conjugated guanine tract that recovers the formation of a damaged G4 consists of two parts [11]: a short guanine tract containing the sequence GGG, which enables it to form an intermolecular G4 structure with a target, and a ligand moiety that stabilizes intermolecular G4 by stacking with the formed G-quartet (Figure 1). Previously, we designed a short (6-mer) oligonucleotide (5′ PyUGGGTT-3′), termed PyG3, containing the 5′-terminal 5-(1-pyrenylethynyl)-2′-deoxyuridine (PyU) conjugate and demonstrated that PyG3 formed a stable intermolecular G4 with damaged VEGF G4 containing 8-oxoG [11]. In this study, we designed a series of new ligand-conjugated guanine tracts (Figure 2A). Planar polyaromatic hydrocarbons of different number of benzene rings such as pyrene, anthracene, and perylene were used as ligand moieties. Pyrene [14], anthracene [15], and perylene [16] are known as G4 binding ligands. We tested two types of pyrene derivatives: one with a shorter linker between the guanine tract and the pyrene residue based on (*S*)-2,4-dihydroxybutyramide backbone, termed PyS [17], and the other with a longer linker based on L-homoserine, termed PyL [18]. Because PyG3 possesses an ethynyl linker between the pyrene residue and the C5 position of the uracil base, we also obtained derivatives of 1-phenylethynylpyrene, termed PEPy, and 9,10-bis (phenylethynyl) anthracene, termed BPEA [19]. For the perylene type, a *trans*-4-hydroxy-L-prolinol derivative with the perylene moiety on a short linker, termed Per, was used. Five controlled-pore glass (CPGs) supports incorporating each ligand-containing linker were prepared (Figure 2A) to introduce the ligand at either the 3′ or 5′ end of the guanine tracts (Figure 2B). Ten ligand-conjugated guanine tracts were prepared by automated oligonucleotide synthesis using conventional 3′-deoxynucleoside β-cyanoethyl phosphoramidites for the 3′-conjugated tracts and the reverse 5′-nucleoside β-cyanoethyl phosphoramidites for the 5′-conjugated tracts (Figure 2B). All the chromatograms and MS spectra are shown in Supporting information Figure S1–S11.

We used the VEGF G2T sequence derived from a VEGF G4 sequence carrying a thymidine residue at the second position within the 5′-terminal guanine tract (Table 1) as target DNA for the genetic mutation model, which depicted oxidative damage to a guanine base. The introduction of 8-oxoG modifying the guanine in the middle quartet has the largest destabilizing effect on the G4 structure [11]. Thus, the G2T mutation caused significant destabilization of the VEGF G4 structure (data not shown), as well as the oxidized VEGF G4s, as observed previously [11].

### 2.2. Structures of Intermolecular G4s of Mutated VEGF with Ligand-Conjugated Guanine Tracts

**Figure 3:**
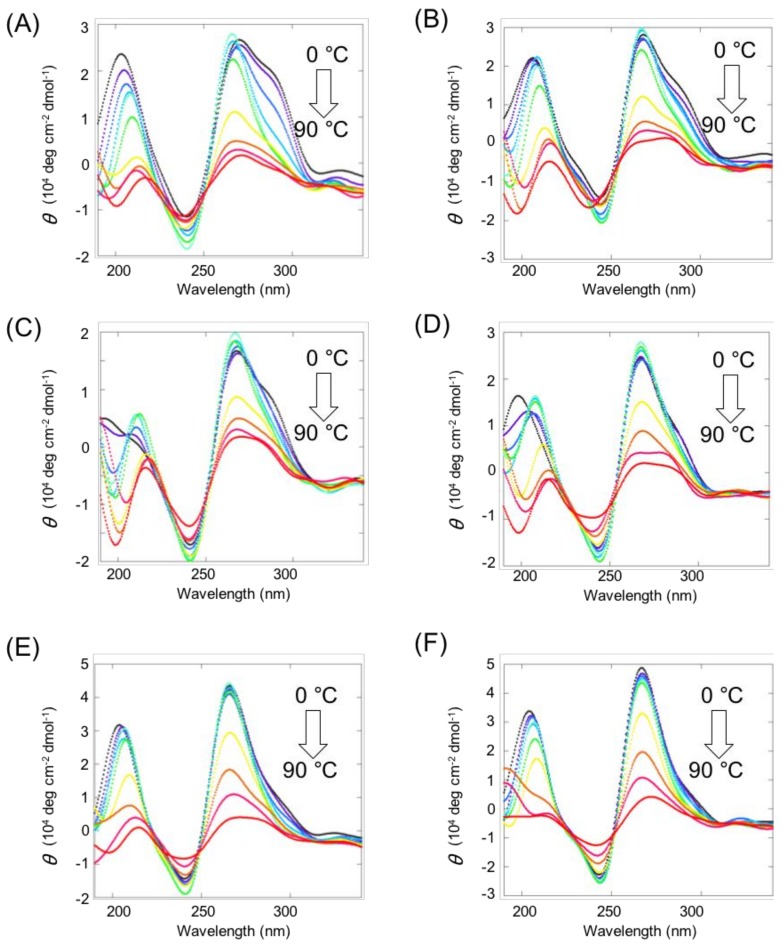
Circular dichroism (CD) spectra of 10 µM VEGF G2T with 10 µM (**A**) PySG_3_, (**B**) G_3_PyS, (**C**) PyLG_3_, (**D**) G_3_PyL, (**E**) PEPyG_3_, and (**F**) G_3_PEPy. All measurements were conducted in a buffer consisting of 10 mM Tris-HCl (pH 7.5), 8 mM MgCl_2_, and 50 mM KCl.

CD spectra were obtained to confirm formation of intermolecular G4s by each guanine tract. CD spectra of 10 µM VEGF G2T complexed with 10 μM ligand-conjugated guanine tract were measured by heating the complex from 0 °C to 90 °C in 10 °C steps in a buffer consisting of 10 mM Tris-HCl (pH 7.5), 8 mM MgCl_2_, and 50 mM KCl (Figure 3). The CD spectrum of VEGF G2T with PySG_3_ at 0 °C showed a large positive peak at 263 nm and a shoulder peak at 295 nm (Figure 3A). The major band at 263 nm was indicative of a typical parallel topology of G4 structures of VEGF G4 (Figure S12), suggesting that intermolecular G4 formation mainly conforms to a parallel topology. The minor band at 295 nm, observed in VEGF G4 with 8-oxoG at the G2 position indicates a topology different from the parallel type. This minor band disappeared when the temperature increased above 30 °C. Thus, under physiological conditions corresponding to 37 °C, the complex forms a parallel topology observed in native VEGF G4 at 37 °C. CD spectra of the G_3_PyS complex were similar to that of the PySG_3_ complex (Figure 3B). PyLG_3_ and G_3_PyL complexes exhibited sharper bands at 295 nm compared to that of PySG_3_ (Figure 3C,D), while those of PEPyG_3_ and G_3_PEPy complexes were much sharper (Figure 3E,F). PyL and PEPy contain linkers which are longer than that of PyS, suggesting that the shorter pyrene linker may not be adequate to form a stable intermolecular G4 in parallel topology, exhibiting a different, non-parallel topology. Enhanced formation of the parallel topology by PEPy-conjugated guanine tracts is attributed to stabilization between the ethynyl group and the G-quartet.

**Figure 4:**
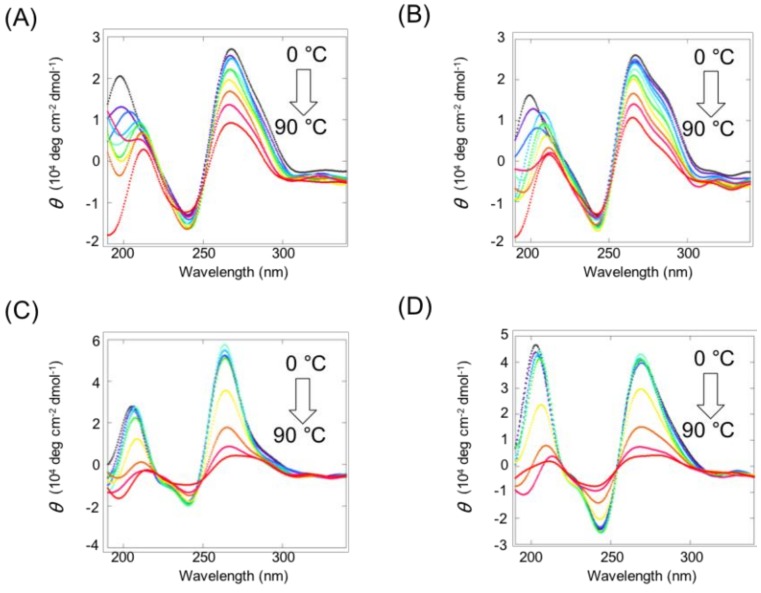
CD spectra of 10 µM VEGF G2T with 10 µM (**A**) BPEAG_3_, (**B**) G_3_BPEA, (**C**) PerG_3_, and (**D**) G_3_Per. All the experiments were conducted in a buffer consisting of 10 mM Tris-HCl (pH 7.5), 8 mM MgCl_2_, and 50 mM KCl.

With regard to BPEA derivatives, the CD spectra of the complex of VEGF G2T, BPEAG_3_, and G_3_BPEA displayed a maximum band around 263 nm (Figure 4A,B). However, the decrease in CD intensity with increasing temperature was not significant, indicating that the interaction between guanine tracts with BPEA and VEGF G2T was insufficient to form intermolecular G4s. On the other hand, the VEGF G2T, PerG_3_, and G_3_Per complex represented CD spectra of a typical parallel G4 (Figure 4C,D). The clear profile of changes in CD spectra between low (<40 °C) and high temperatures (>80 °C) suggests that the formed intermolecular G4 structures had dissociated due to increasing temperatures. Thus, the quality of intermolecular G4s also depends on the structure of the conjugated ligand.

### 2.3. Stability of Intermolecular G4s of Mutated VEGF with Ligand-Conjugated Guanine Tracts

**Figure 5:**
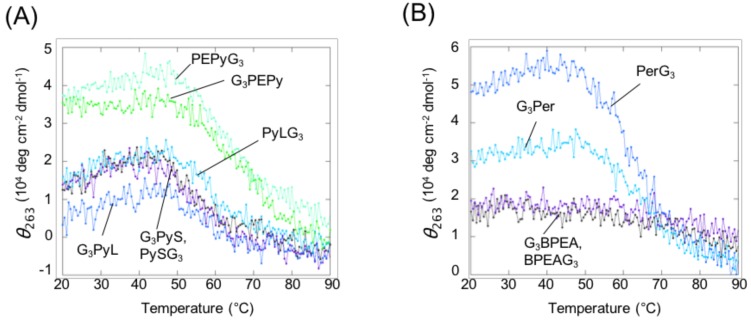
CD melting profiles of 10 µM VEGF G2T with (**A**) pyrene-conjugated guanin tracts including PySG_3_ (**black**), G_3_PyS (**purple**), PyLG_3_ (**light blue**), G_3_PyL (**blue**), PEPyG_3_ (**pale green**), and G_3_PEPy (**green**), and (**B**) anthracene or perylene derivatives including BPEAG_3_ (**black**), G_3_BPEA (**purple**), PerG_3_ (**light blue**), and G_3_Per (**blue**). CD melting data were collected by measuring CD intensity at 263 nm in a buffer consisting of 10 mM Tris-HCl (pH 7.5), 8 mM MgCl_2_, and 50 mM KCl.

Next, changes in CD intensity at 263 nm were tracked to analyze stability of the VEGF G2T complex with each ligand-conjugated guanine tract based on the two-state transition model. Except for BPEA derivatives, all complexes exhibited a sigmoidal pattern of CD intensity decrease with increasing temperatures (Figure 5). The thermodynamic parameters of the intermolecular G4 formation of 10 µM VEGF G2T with the 10 μM ligand-conjugated guanine tract complex and 50 mM KCl are shown in Table 2. PyS derivatives exhibited the lowest stability, whereas PEPy derivatives had a higher stability compared to both PyS derivatives as well as Per derivatives. The Per derivative has the largest fused aromatic structures, which contributes to the relative high stability of the intermolecular G4 with Per type guanine tracts, despite the relatively short and rigid linker of both 3′- and 5′-Per derivatives. In contrast, the ends to which the ligand attaches have no apparent effect on stability, although G_3_Per displayed somewhat higher stability compared to that of PerG_3_. As previously mentioned, in the presence of 10 µM PyG3, 10 µM VEGF with 8-oxoG at the second guanine (VEGF G2O) exhibited a *T*_m_ of 66.9 °C and −Δ*G*°_37_ of 4.4 kcal mol^−1^, respectively [11], which indicated slightly greater stability compared to other constructs tested in this study. This difference may be due to certain favorable interactions between the pyrene moiety and 8-oxoG, compared to the absence of any interaction between each ligand moiety and thymine.

### 2.4. Replication Assay of Mutated VEGF G4 with Ligand-Conjugated Guanine Tracts

**Figure 6:**
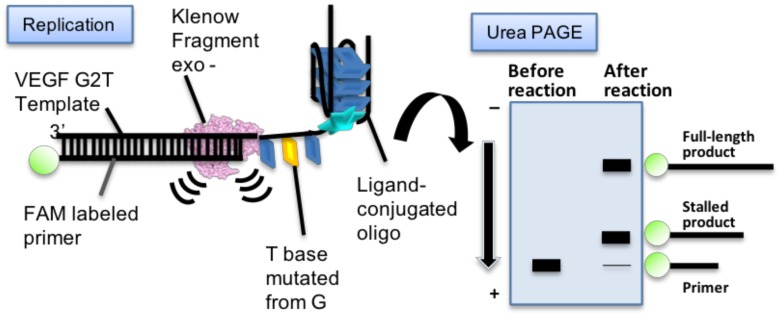
Schematic illustration of the replication assay.

In order to test the biological function of G4 recovered by ligand-conjugated guanine tracts, a replication assay was performed. A fluorescein-labeled primer and template DNA containing a VEGF G2T sequence (Table 1) was designed for this replication assay as reported previously (Figure 6) [11]. The replication reaction was carried out using these DNAs and the Klenow fragment DNA polymerase lacking 3′→5′ exonuclease activity (KF exo-) at 37 °C in the same buffer solution used for the CD experiments. Replication stalling efficiency of template DNA at G4 was analyzed by denaturing polyacrylamide gel electrophoresis (PAGE; as shown in Figure S13 in the case of native VEGF G4) of reaction samples obtained at each time point.

**Figure 7:**
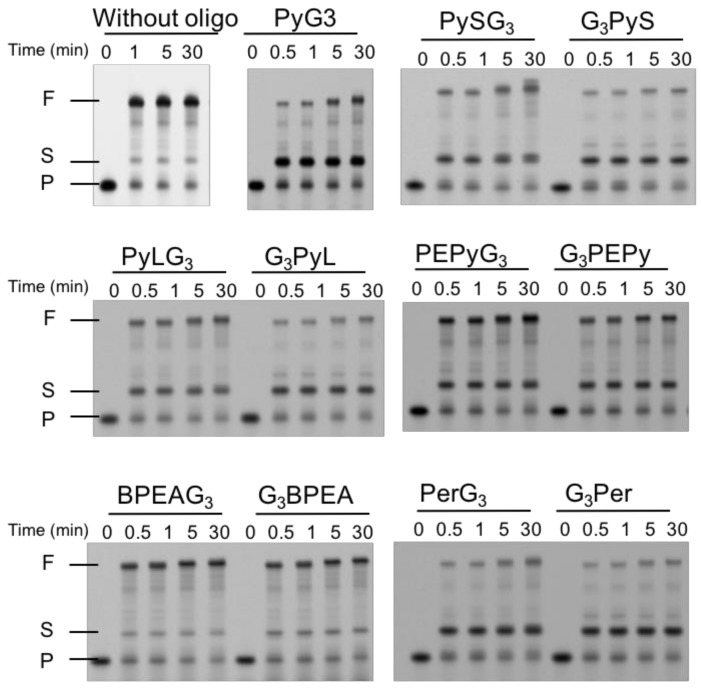
Denaturing-PAGE images of replication products from the VEGF G2T template with each ligand-conjugated guanine tract. All assays were performed in a buffer consisting of 10 mM Tris-HCl (pH 7.5), 8 mM MgCl_2_, and 50 mM KCl with 1 µM primer, 1 µM template, 10 µM ligand-conjugated guanine tract, 250 µM dNTPs, and 1 µM KF exo- at 37 °C.

In the absence of the guanine tract, replication products showed far fewer stalled products, and exhibited instant accumulation of full-length products within 1 min following initiation of the reaction (Figure 7). This result was similar to that of the oxidized VEGF G4 replication assay reported previously [11]. In contrast, PyG3 stalled replication at the 3′-end of the G4 recovered from VEGF G2T (Figure 7). The stalled product was detectable as early as 30 min following reaction initiation. These results were consistent with previous reports using oxidized VEGF G4s (Figure 7) [11]. In the presence of new ligand-conjugated guanine tracts, PAGE analyses revealed accumulation of short products similar to that of PyG3, indicating that the replication complex stalled on G4 recovered from VEGF G2T in all cases except in the one with BPEA derivatives (Figure 7). To compare replication efficiencies, the ratio of full-length products was analyzed against all extended primers following 30 min reactions. The results show that replication efficiency differs depending on each ligand and attached end. Regarding PyS derivatives, full-length products were suppressed to a higher level in the presence of G_3_PyS (24.9%) than in the presence of PySG_3_ (45.3%). As described in the previous section, the stabilities of VEGF G2T with guanine tracts of the two PyS derivatives were quite similar to each other. Thus, replication of recovered VEGF G4 was regulated by not only stability of the complex with the guanine tract, but also by its structure. The effect of the position of attachment on replication stalling was seen in all other conjugated ligands with the stalled product ratio as follows; PyLG_3_ (42.9%), G_3_PyL (27.7%), PEPyG_3_ (46.4%), G_3_PEPy (36.9%), PerG_3_ (31.6%), and G_3_Per (20.2%). The order of replication suppression also differed for different ligand molecules. PyS derivatives produced similar results to PEPy derivatives. PyL derivatives suppressed replication with similar efficiency to PyS, while Per derivatives exhibited the highest level of suppression among the ligands studied. The association between the ligand moiety and replication stalling cannot be attributed to the stability of the formed intermolecular G4s, as all derivatives except those of PyS showed similar stabilities. Therefore, these results suggest that the efficiency of replication suppression by ligand-conjugated guanine tracts depends on the unfolding mechanism of intermolecular G4s by DNA polymerase, which varies according to the mechanism of interaction between the ligand moiety and the mutated G4.

## 3. Discussion

**Figure 8:**
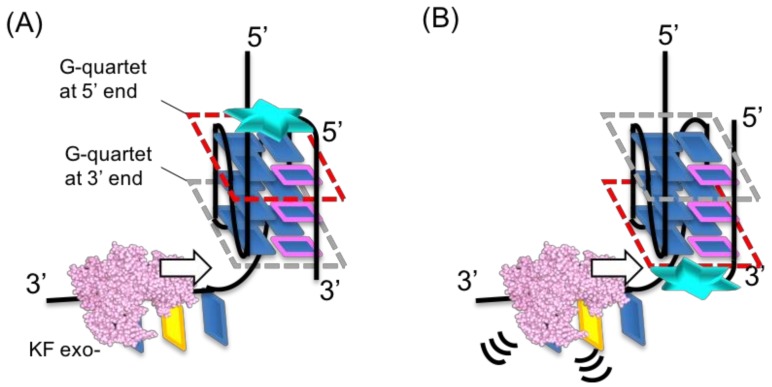
Regulation of G4 replication by a guanine tract conjugated at its (**A**) 5′ end and (**B**) 3′ end. The boxes highlighted in red indicate the affected G-quartet with the ligand moiety to decrease the unfolding kinetics.

Intermolecular G4 formation with a ligand-conjugated guanine tract depends on the interaction between the ligand moiety and the G4 structure. Our study conducted a systematic evaluation of the structure of the ligand moiety and its position of attachment on the guanine tract. Our results indicate that for replication suppression, all tested guanine tracts preferred attachment at the 3′ end rather than at the 5′ end. The CD analysis results imply that intermolecular G4s form a parallel topology (Figure 3 and Figure 4), indicating that the surface of interaction between the ligand and G-quartet on G4 varied depending on the end at which it was attached. A G-quartet in G4s possesses two surfaces for interacting with small molecules (Figure 8). As the parallel topology of G4 forms propeller loops, both surfaces of the top and bottom G-quartet are exposed to the solvent, as opposed to other topologies that have diagonal and lateral loops, which may interact with G-quartet surfaces. Both G-quartet surfaces in a parallel topology are equivalent. Thus, most G4 ligands interact with G4 exhibiting a parallel topology in a 2:1 upper to lower surface ratio [20]. In this study, the ligand-conjugated guanine tract was replaced with the first guanine tract of VEGF G4. Based on this assumption, the ligand attached to the 5′-end of the guanine tract should bind to the G-quartet surface facing the 3′-end of VEGF G4, and the ligand attached to the 3′-end should bind to the opposite surface (Figure 8). Both intermolecular G4s formed with each ligand showed similar stability, corresponding to equivalent surfaces of each G-quartet. Thus, the difference in the replication suppression is due to the polarity of the replication reaction, where DNA polymerase catalyzes DNA polymerization along a template DNA from its 3′-end to 5′-end. In duplex DNAs, fraying of the terminal A-T base pair is larger than that of the terminal G-C base pair [21], indicating that the unfolding kinetics of a terminal base pair correlates with its stability. This property of DNA ends implies that a ligand-conjugated guanine tract can specifically decrease the unfolding kinetics of either end of G4, by switching the attached end of the ligand moiety (Figure 8). A ligand conjugated at the 5′-end of a guanine tract may not reduce the unfolding kinetics of the G4 from the 5′-end, thus not affecting replication processivity (Figure 8A). On the other hand, a ligand conjugated to the 3′-end of guanine tracts may decrease the unfolding kinetics compared to that of the 5′-end of a G4, thus inhibiting replication processivity (Figure 8B). Considering that available information related to the effects of G4 ligands on replication is scarce, we safely assume that our study is the first to demonstrate regulation of replication along the G4 by the polarity of DNA polymerase processivity.

The ethynyl group in the linker of PEPy derivatives increased the stability to a larger extent than PyS and PyL derivatives. This may be due to additional interaction between the ethynyl group and the G-quartet based on π–π or CH–π interactions [22]. Nevertheless, the replication stalls by PEPy derivatives were not more significant than those in PyS and PyL derivatives. This result indicates that the stabilization effect by the ethynyl group does not interrupt the unfolding kinetics of the G4 structure by DNA polymerase. Thus, not only the aromatic moiety of ligands, but also the linker moiety can control the function of the ligand-conjugated guanine tract.

Since G4 formation in the promoter region of some cancer genes inhibits gene transcription [1], stabilization of G4s can contribute to cancer therapeutics. However, a stabilized G4 obstructs replication, leading to undesirable genetic instability followed by gene mutations [23]. The results of this study indicate that guanine tracts conjugated at the 5′ end may decrease the risk of a replication stall at the formed G4. Therefore, a technique focused on the structural polarity of G4 would provide a novel strategy for recovering the formation of mutated G4s and tuning associated reactions, such as replication and transcription.

## 4. Materials and Methods

### 4.1. Materials

dNTPs were purchased from Takara Bio (Shiga, Japan). Ligands were dissolved in Milli-Q water or dimethyl sulfoxide. Other reagents were purchased from Wako Pure Chemical Industries (Osaka, Japan) and used without further purification.

### 4.2. Oligonucleotides

All DNA sequences used in this study for replication assays are listed in Table 1. Oligonucleotide PyG3 was synthesized as reported previously [11]. HPLC-purified fluorescein (FAM)-labeled primer and template DNAs were purchased from Japan Bio Service (Saitama, Japan). Ligand-conjugated oligonucleotides were synthesized on an automated DNA/RNA synthesizer ASM-800 (Biosset, Novosibirsk, Russia) on a scale of 0.4 μmol using conventional 5′-dimethoxytrityl (DMTr) N^2^-isobutyryl-dG and T 2-cyanoethyl-N,N-diisopropyl 3′-phosphoramidites (Sigma-Aldrich, St. Louis, MO, USA), and the respective reverse 3′-DMTr 5′-phosphoramidites (ChemGenes, Wilmington, MA, USA). Oligonucleotides were assembled in the ‘DMTr off′-mode according to standard protocols supplied by manufacturer. After completion of the synthesis, oligonucleotides were cleaved from solid support and deprotected by a 1:1 (*v*/*v*) mixture of 25% aqueous ammonia and 40% aqueous methylamine (AMA) for 15 min at 55 °C. After removal of the volatiles in vacuo, oligonucleotides were dissolved in MilliQ water and subjected to HPLC analysis and purification as described in the following paragraph.

Analytical reverse-phased (RP) HPLC was performed in an Agilent 1220 system (Agilent Technologies, Santa Clara, CA, USA) with UV detection at 260 nm using a ZORBAX Eclipse XDB-C18 5 μm 4.6 × 150 mm column (Agilent Technologies) eluted with a 0–60% gradient of acetonitrile (eluent B) in 20 mM triethylammonium acetate (TEAA), pH 7.0 (eluent B) for 30 min, flow rate 1 mL/min. A semi-preparative isolation of oligonucleotides was carried out on a Waters 600E chromatograph (Waters, Milford, MA, USA) with UV detection at 190 nm, 260 nm and 280 nm using a ZORBAX Eclipse PrepHT XDB-C18 column (7 μm, 21.2 × 150 mm, Agilent Technologies) and the same gradient of eluent B in eluent A at flow rate of 21 mL/min. Fractions containing ligand-conjugated oligonucleotides were collected and freeze-dried. Oligonucleotide concentration was calculated by measuring the A_260_ of oligonucleotide solutions on a NanoDrop 2000c UV-Vis spectrophotometer (ThermoFisher Scientific, Waltham, MA, USA). All the chromatograms are shown in the supporting information.

Molecular masses of oligonucleotides were determined by MALDI-TOF mass spectrometry on a Reflex III Autoflex Speed spectrometer (Bruker, Bremen, Germany) with 2,6-dihydroxyacetophenone–diammonium citrate as a matrix in the negative ion detection mode using standard device settings. Molecular masses of the oligonucleotides were calculated using sets of experimental *m*/*z* values, which were evaluated for each sample. All the MS spectra are shown in the supporting information.

CPG supports for solid-phase oligonucleotide synthesis loaded with the corresponding ligand-containing linkers PyS (96.1 µmol/g), PyL (60.1 µmol/g), PEPy (44.1 µmol/g), BPEA (35.0 µmol/g), and Per (29.9 µmol/g) were obtained according to guidelines in the literature [24]. The PyS linker (N-(1-pyrenemethyl)-(*S*)-2,4-dihydroxybutyramide) was synthesized as described previously [17,25]. The PyL linker was prepared as described in reference [18]. PEPy and BPEA linkers were likewise obtained using a previously developed method [19]. The Per linker (N-(1-peryleneacetyl)-*trans*-4-hydroxy-L-prolinol) was prepared as described in reference [26].

### 4.3. CD Spectrum Acquisition

For CD spectroscopy, 10 μM VEGF G2T with and without 10 μM of each ligand-conjugated guanine tract was added to the buffer consisting of 10 mM Tris-HCl (pH 7.5), 8 mM MgCl_2_, and 50 mM KCl. Samples were heated to 95 °C for 3 min and cooled to 0 °C at a rate of −1.0 °C min^−1^. Then, the temperature was increased from 0 °C to 90 °C at a rate of 0.5 °C min^−1^. CD spectra were recorded using a JASCO J-1500 at 10 °C intervals in the range from 0 °C to 90 °C.

### 4.4. CD Melting Assay

For melting assays, 10 μM VEGF G2T, with and without 10 μM of each ligand-conjugated guanine tract was added to the buffer consisting of 10 mM Tris-HCl (pH 7.5), 8 mM MgCl_2_, and 50 mM KCl. Melting analyses were performed using a JASCO J-1500 equipped with a temperature control system. Samples were cooled from 90 °C to 0 °C at a rate of −1.0 °C min^−1^. Then, the temperature was increased from 0 °C to 90 °C at 0.5 °C min^−1^. CD melting data were collected by measuring CD intensity at 263 nm. In order to determine the thermodynamic parameters, CD melting curves were normalized and analyzed by curve fitting using Kaleida Graph software (version 4.5, Synergy Software, Reading, PA, USA). We conducted this assay three times for each sequence.

### 4.5. Replication Assays

The Klenow fragment (exo-) was prepared as described [11]. Before addition of KF exo-, the primer and template DNA were annealed in the replication reaction buffer (10 mM Tris-HCl (pH 7.5), 8 mM MgCl_2_, 1 μM KF exo-, 1 μM template DNA, 50 mM KCl, and 250 μM dNTPs) in the presence of 10 μM ligand-conjugated guanine tract. After preparing the solution, the mixtures were incubated at 37 °C. The reaction was quenched with the addition of 10 mM EDTA and 80% (wt) formamide. Products were separated by PAGE in a gel containing 8 M urea at 200 V and 70 °C for 1 h in TBE buffer. Gel images were captured using a Fluoreimager FLA-5100 (Fujifilm) before and after staining with SYBR Gold (Thermo Fisher Scientific). Band intensities were determined using NIH ImageJ software. The amount of full-length product was quantified using the intensity ratio of the full-length product band compared to all the other detected bands.

## Figures and Tables

**Table 1:** Nucleotide sequences used in this study.

Name	Sequence (5′→ 3′)
VEGF G2T	CAGTGCGGGCCTTGGGCGGGAT
VEGF G2T template	CAGTGCGGGCCTTGGGCGGGATCGGACCTATAGTGAGTCGTATTCCC
FAM labeled primer	[FITC]-GGGAATACGACTCACTATAGG

**Table 2:** Thermodynamic parameters of the VEGF G2T G4 with different ligand-conjugated guanine tracts ^a^.

Ligand-Conjugated Guanine Tract	*T*_m_ (°C)	−∆*G*°_37_ (kcal mol^−1^)
PySG_3_	50.7	2.6 ± 0.4
G_3_PyS	51.6	2.7 ± 0.4
PyLG_3_	53.5	3.3 ± 0.5
G_3_PyL	55.5	3.5 ± 0.5
PEPyG_3_	58.4	3.6 ± 0.6
G_3_PEPy	65.3	3.7 ± 2.1
BPEAG_3_	n.d.	n.d.
G_3_BPEA	n.d.	n.d.
PerG_3_	59.7	3.3 ± 0.8
G_3_Per	62.1	3.7 ± 1.0

^a^ All experiments were performed in 10 μM DNA in a buffer solution consisting of 10 mM Tris-HCl (pH 7.5), 8 mM MgCl_2_, and 50 mM KCl.

## Supplementary Materials

The supplementary materials are available online.
