# Supplementary material for: Design and Properties of Ligand-Conjugated Guanine Oligonucleotides for Recovery of Mutated G-Quadruplexes
Source: Molecules. 2018 Dec 6;23(12):3228. doi: 10.3390/molecules23123228 (PMC6321378; doi:10.3390/molecules23123228)
Supplement: Supplementary file 1 [file molecules-23-03228-s001.pdf]

Supporting Information

Design and properties of ligand-conjugated guanine oligonucleotides for recovery of mutated G-quadruplexes

Shuntaro Takahashi[1], Boris Chelobanov[2,3], Ki Tae Kim[4,#], Byeang Hyean Kim[4], Dmitry Stetsenko[2,3], and Naoki Sugimoto[1,5]*

[1]*Frontier Institute for Biomolecular Engineering Research (FIBER), Konan University, 7-1-20 Minatojima-Minamimachi, Chuo-ku, Kobe, 650-0047, Japan*

[2]*Institute of Chemical Biology and Fundamental Medicine, Siberian Branch of the Russian Academy of Sciences, 8 Lavrentiev Ave., Novosibirsk 630090, Russia*

[3]*Novosibirsk State University, 2 Pirogov Str., Novosibirsk 630090, Russia*

[4]*Department of Chemistry, Division of Advanced Materials Science, Pohang University of Science and Technology (POSTECH), Pohang 37673, Republic of Korea*

[5]*Graduate School of Frontiers of Innovative Research in Science and Technology (FIRST), Konan University, 7-1-20 Minatojima-Minamimachi, Chuo-ku, Kobe, 650-0047, Japan*

* Author to whom correspondence should be addressed; e-mail: sugimoto@konan-u.ac.jp; tel.: +81-78-303-1147; fax: +81-78-303-1495.

# Present Address : Department of Organic Chemistry, Faculty of Science, University of Geneva, 30 quai Ernest Ansermet, 1211 Geneva, Switzerland

Content

Analytical reverse-phased (RP) HPLC profiles and MALDI-TOF mass spectra for the ligand-conjugated guanine tract oligonucleotides **PySG$_3$** (S2), **G$_3$PyS** (S3), **PyLG$_3$** (S4), **G$_3$PyL** (S5), **PEPyG$_3$** (S6), **G$_3$PEPy** (S7), **BPEAG$_3$** (S8), **G$_3$BPEA** (S9), **PerG$_3$** (S10) and **G$_3$Per** (S11). CD spectra of native VEGF G4 (S12). PAGE image of replication assay for native VEGF G4 (S13).

PySG$_3$ = 5’-**X**pGpGpGpTpT-3’

X = 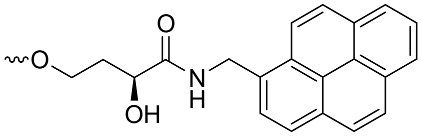

RP-HPLC profile

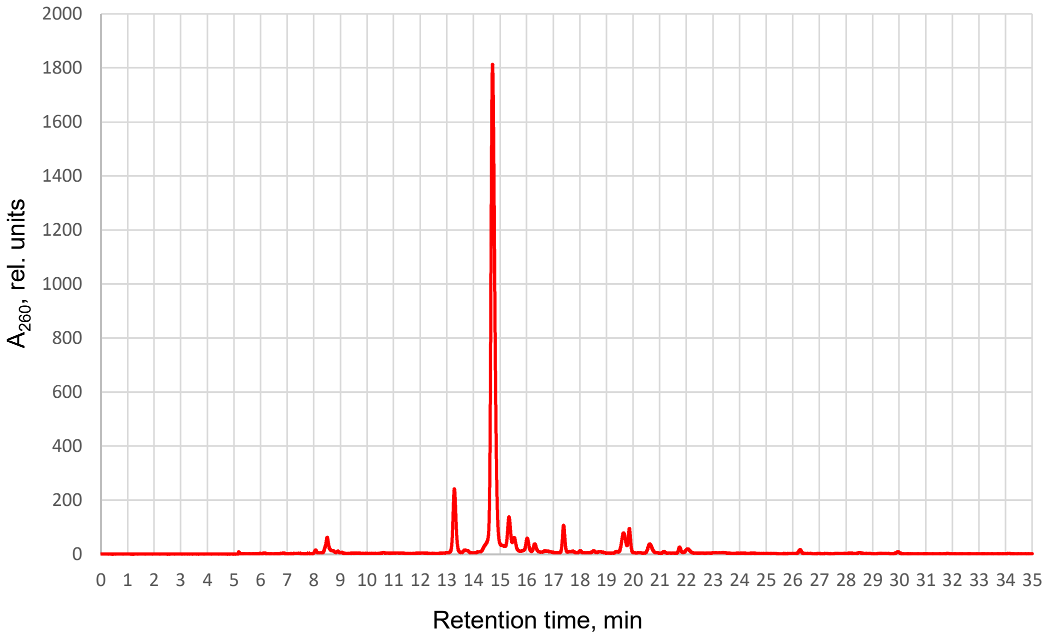

MALDI-TOF MS

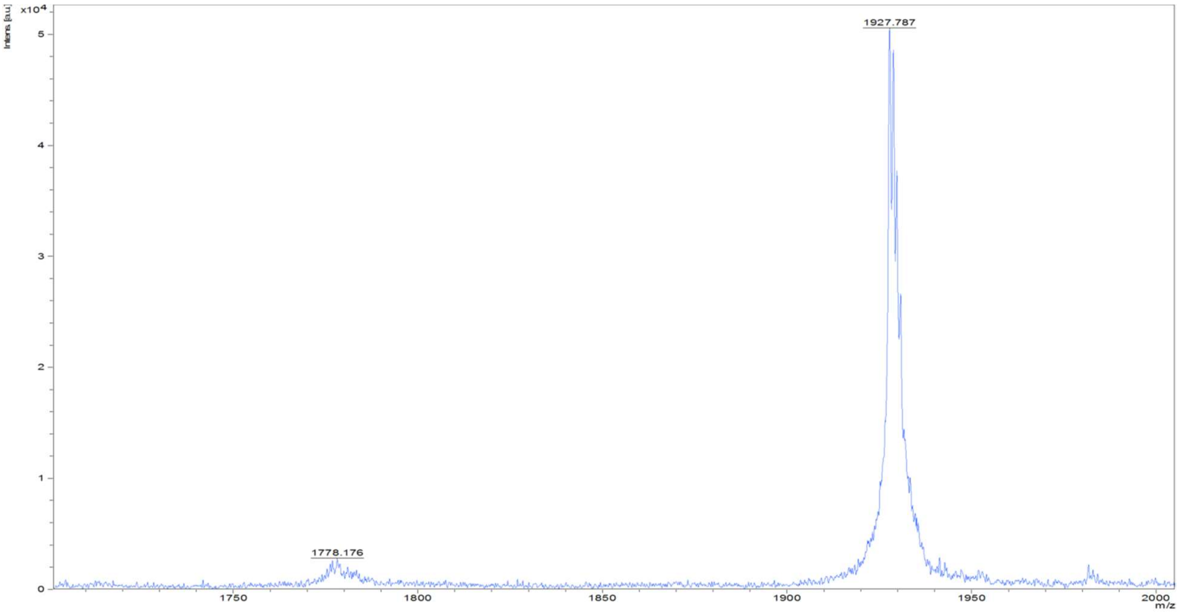

G$_3$PyS = 5’-TpTpGpGpGp**X**-3’

X = 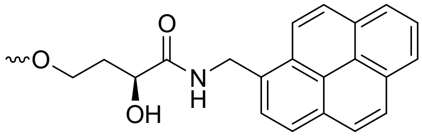

RP-HPLC profile

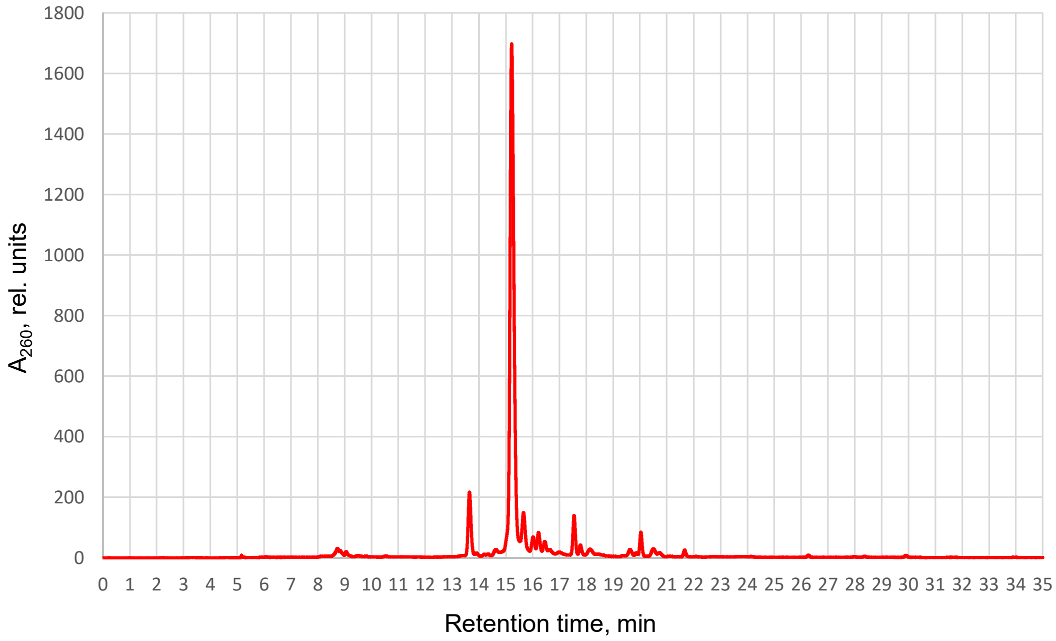

MALDI-TOF MS

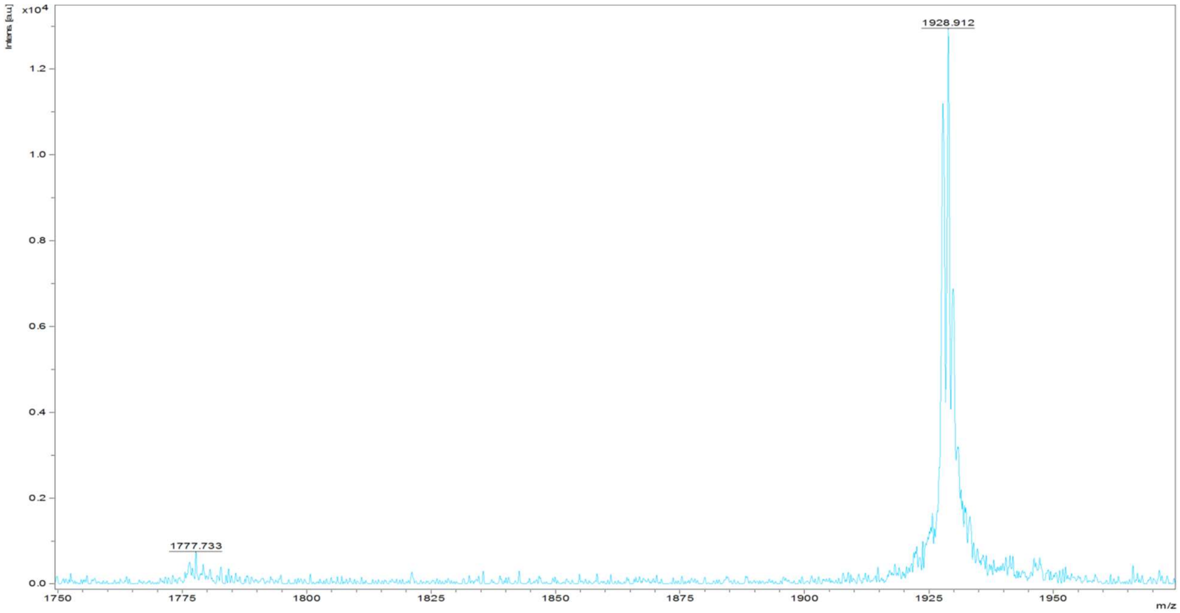

PyLG$_3$ = 5’-**X**pGpGpGpTpT-3’

X =

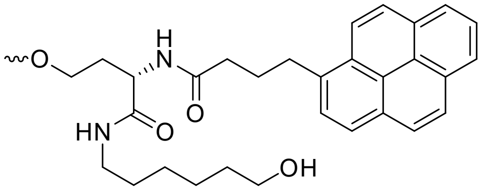

RP-HPLC profile

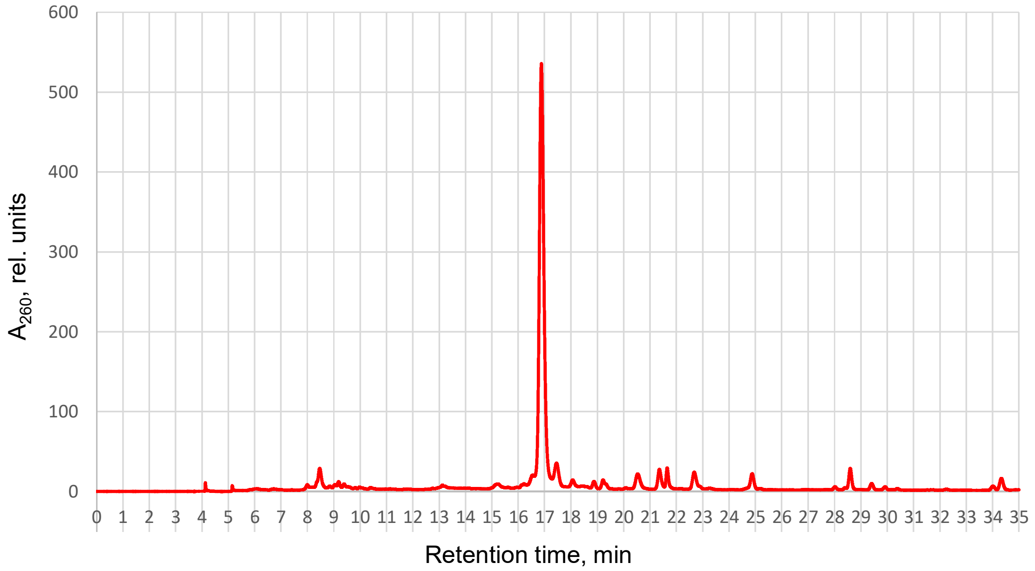

MALDI-TOF MS

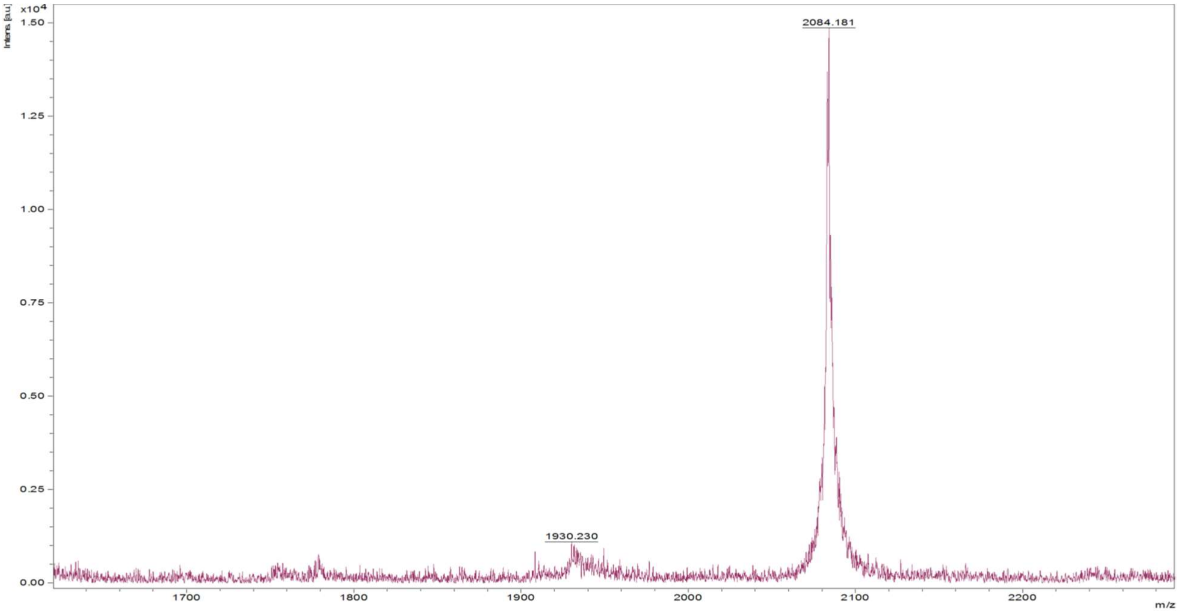

G3PyL = 5’-TpTpGpGpGp**X**-3’

X =

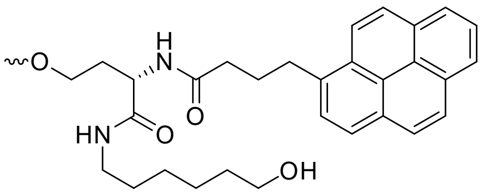

RP-HPLC profile

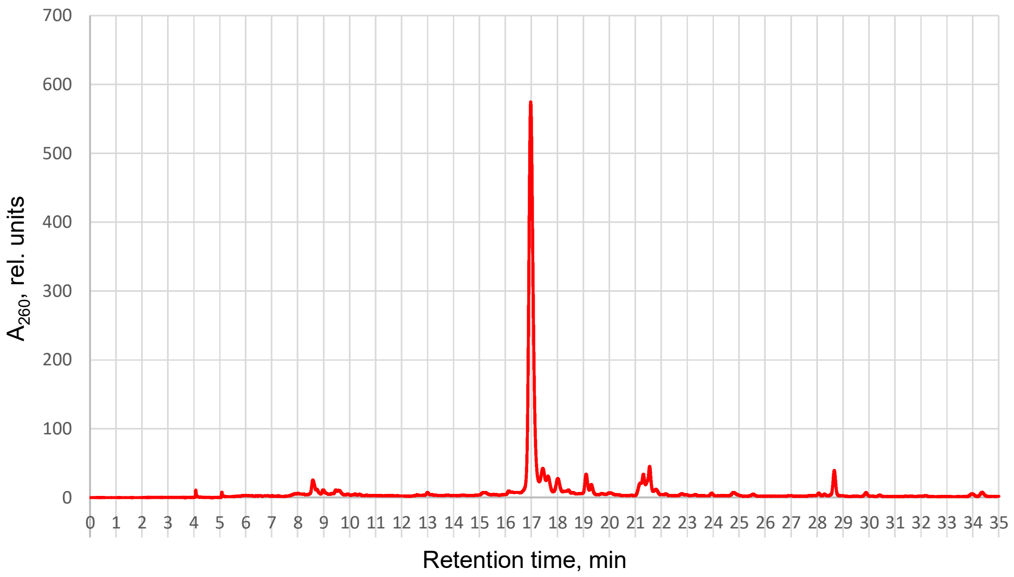

MALDI-TOF MS

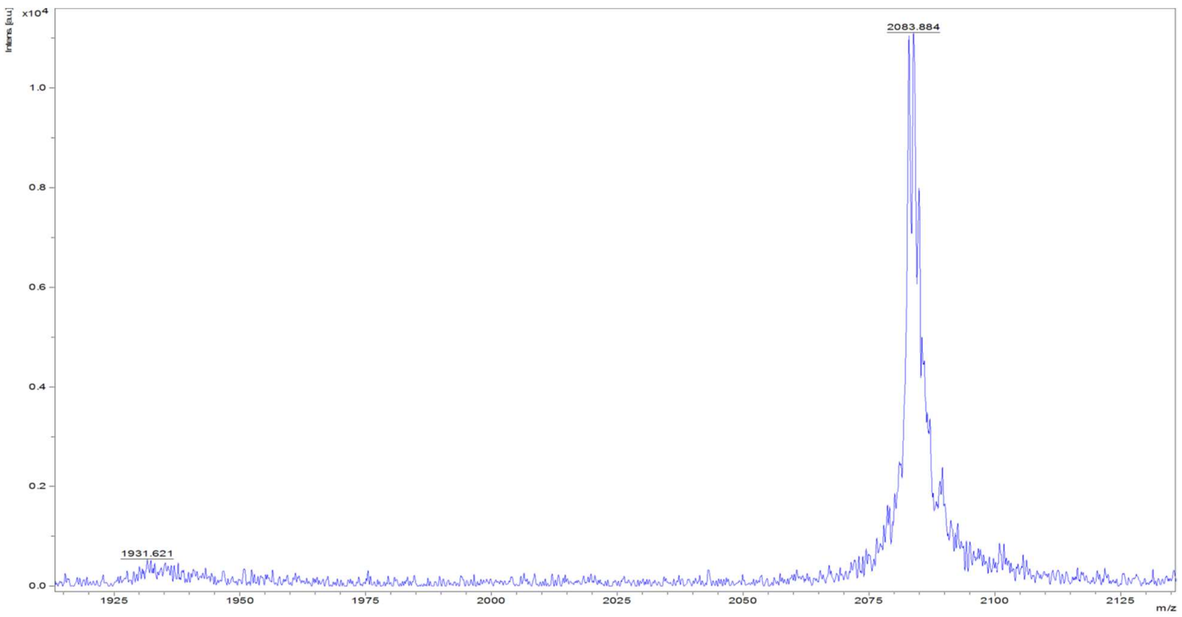

PEPyG3 = 5’-**X**pGpGpGpTpT-3’

X =

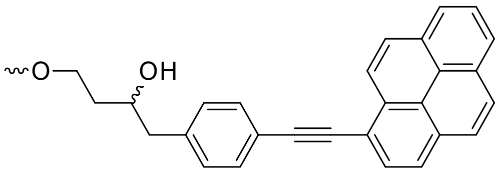

RP-HPLC profile

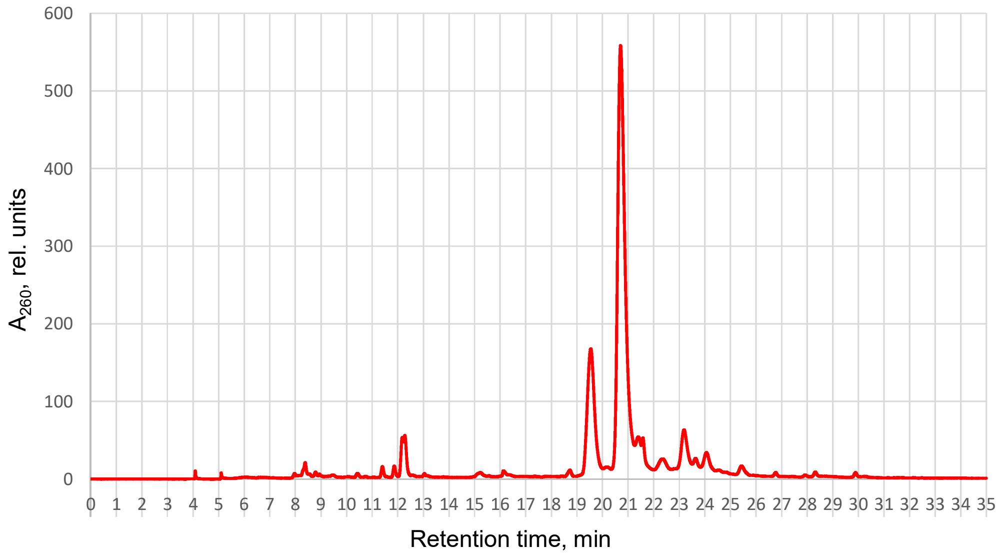

MALDI-TOF MS

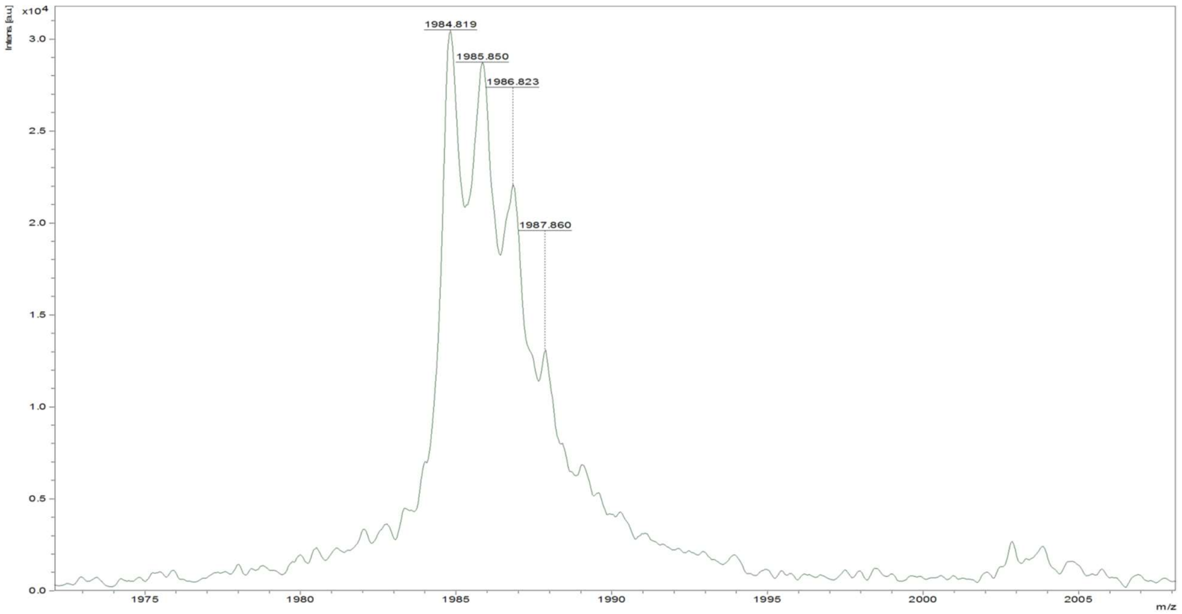

G3PEPy = 5’-TpTpGpGpGp**X**-3’

X =
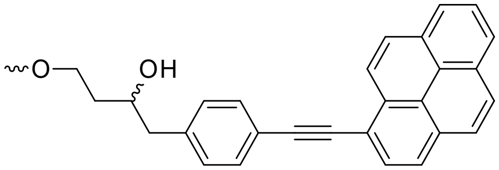

RP-HPLC profile

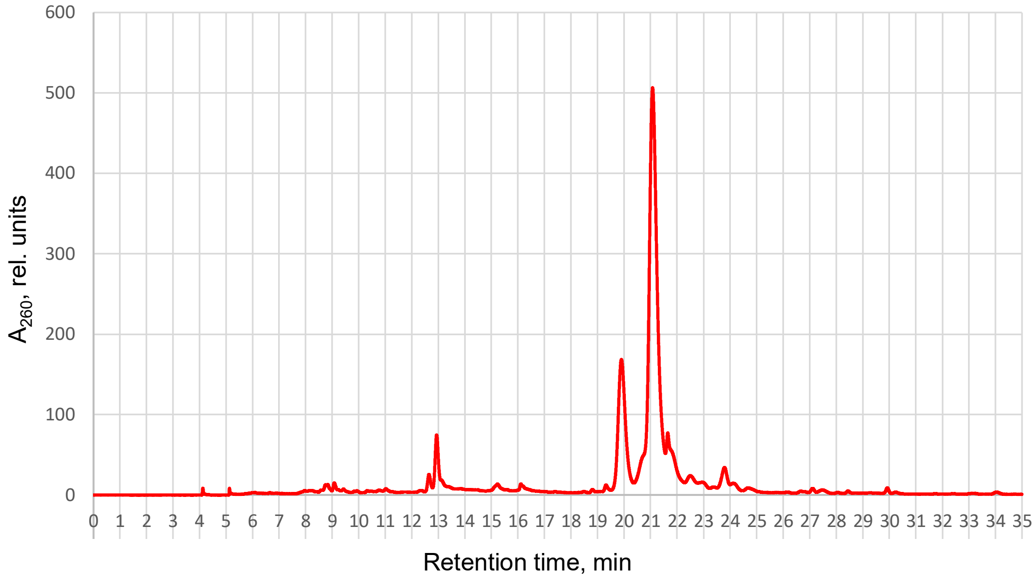

MALDI-TOF MS

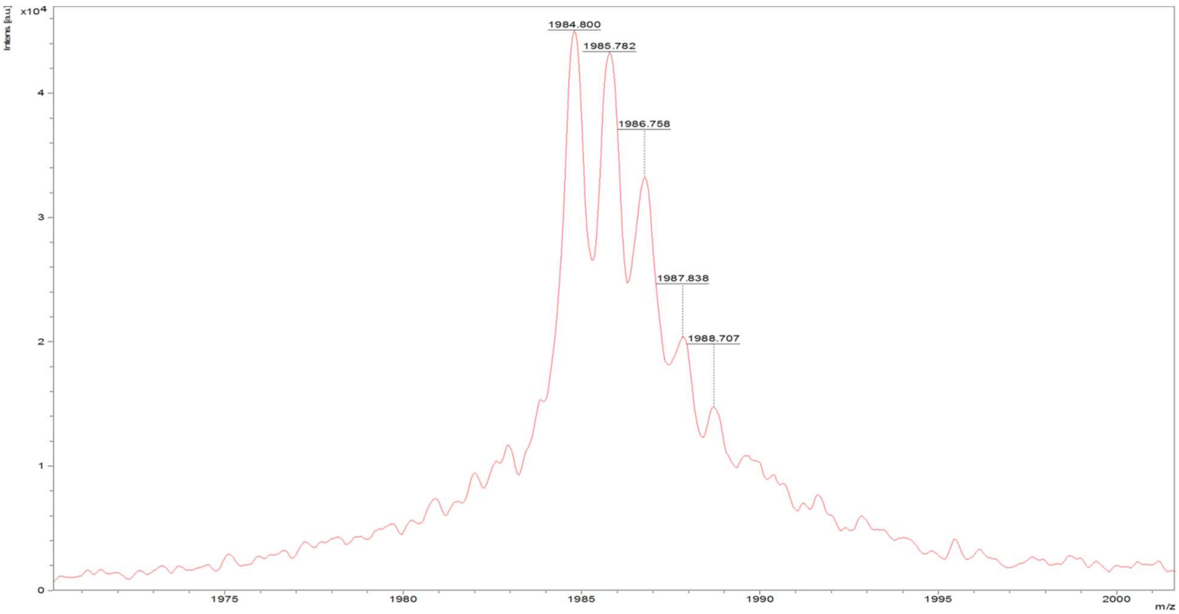

BPEAG3 = 5'-**X**pGpGpGpTpT-3'

X =

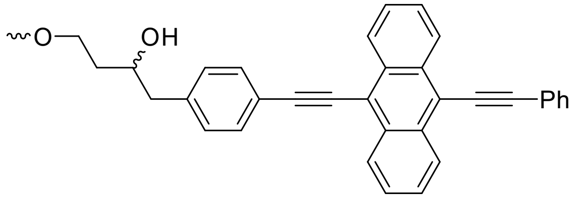

RP-HPLC profile

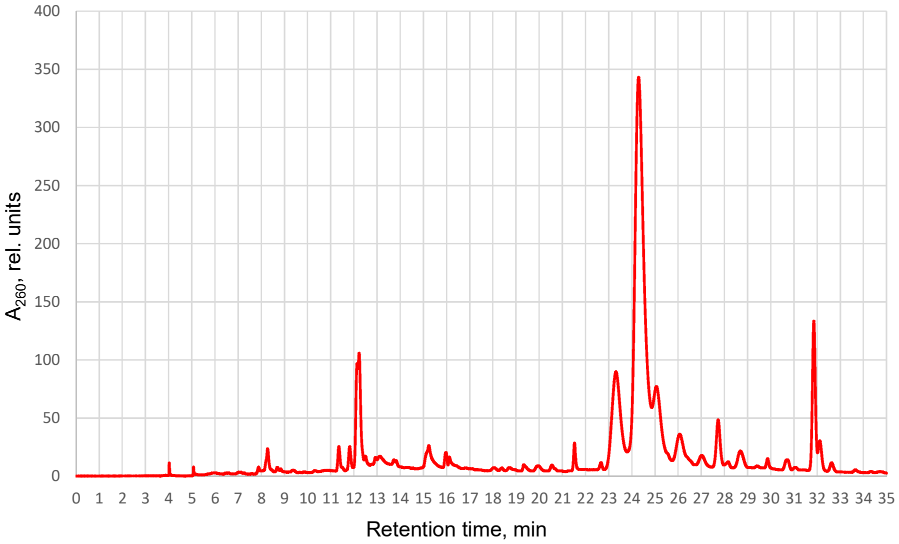

MALDI-TOF MS

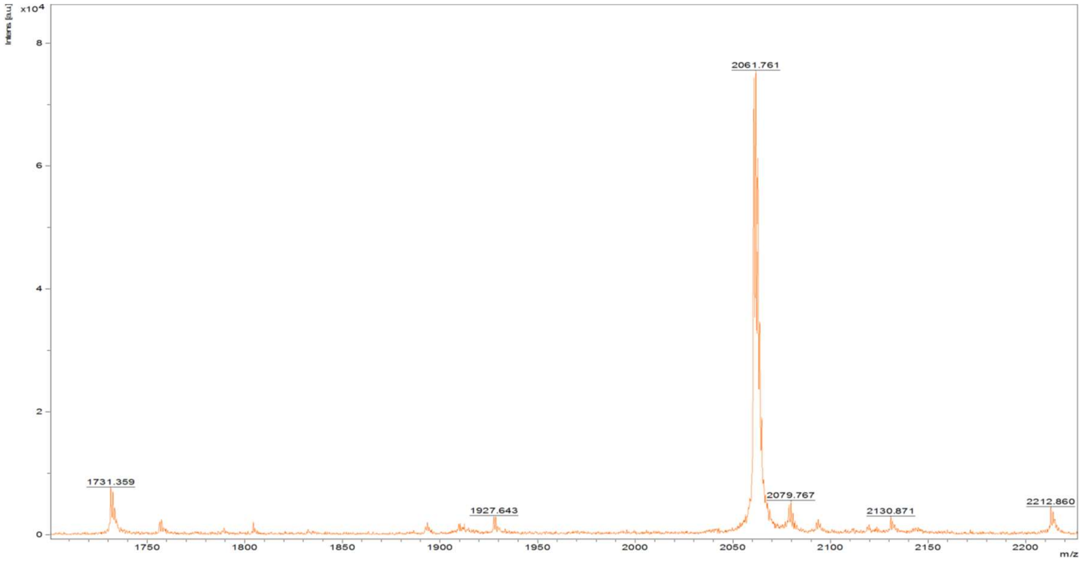

G3BPEA = 5'-TpTpGpGpGp**X**-3'

X =

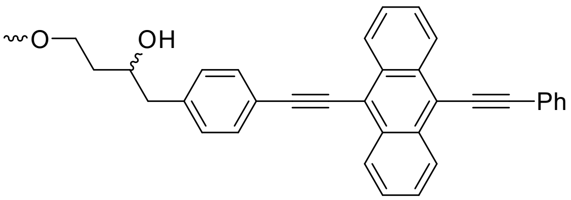

RP-HPLC profile

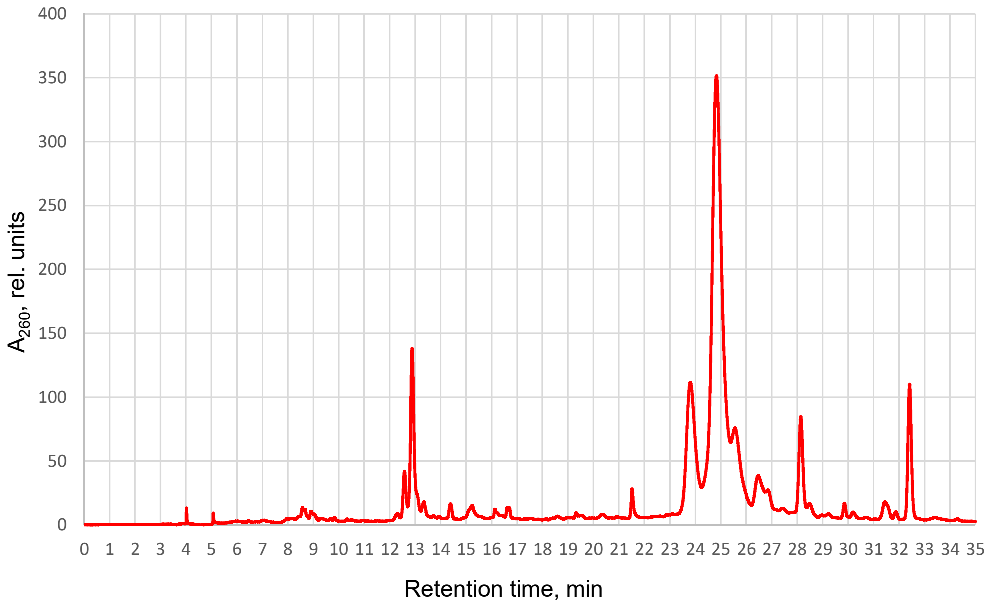

MALDI-TOF MS

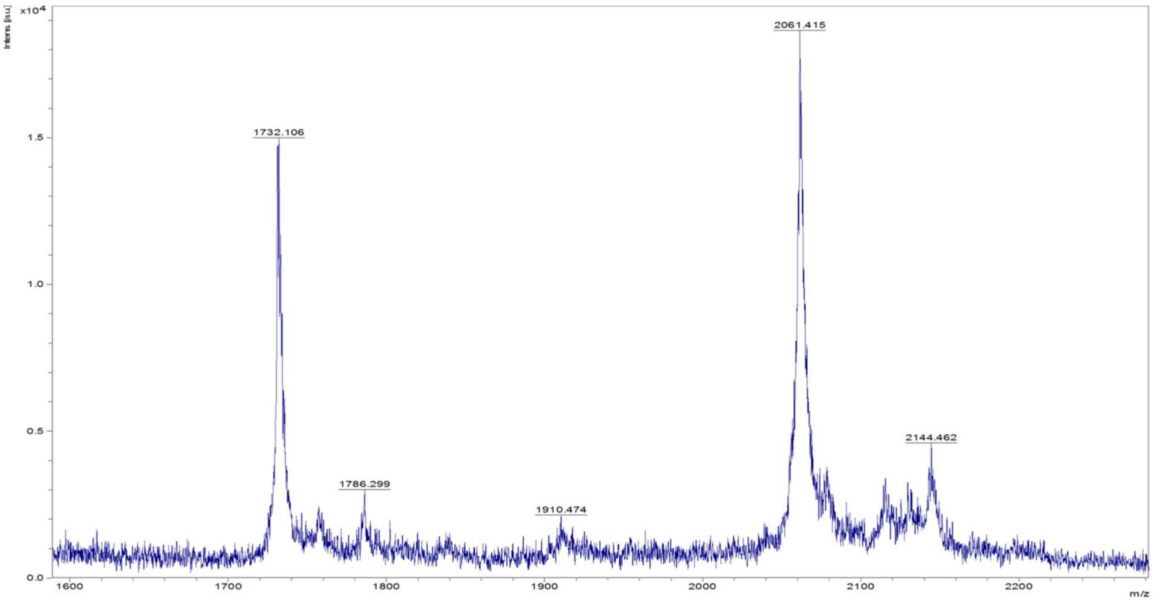

PerG$_3$ = 5'-**X**pGpGpGpTpT-3'

X =

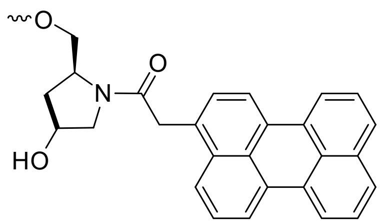

RP-HPLC profile

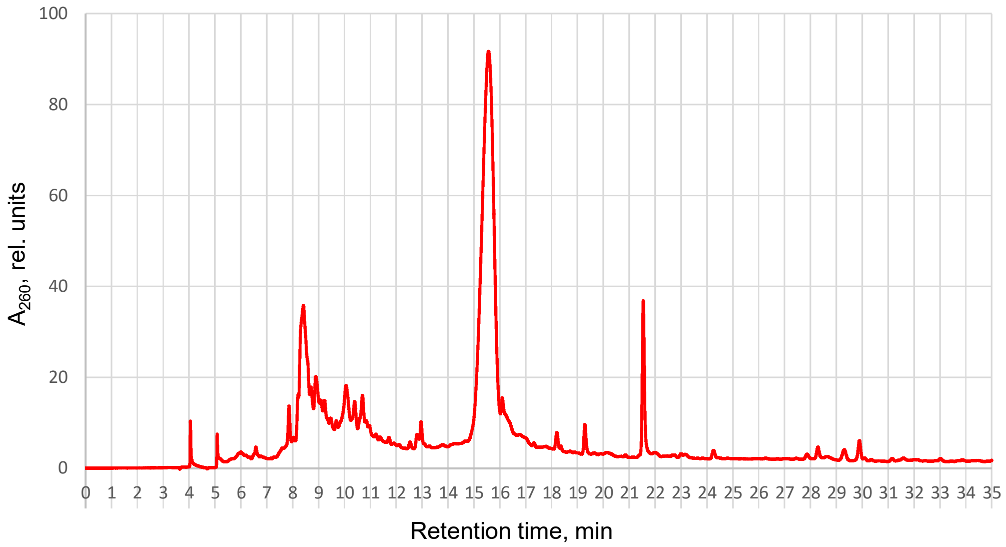

MALDI-TOF MS

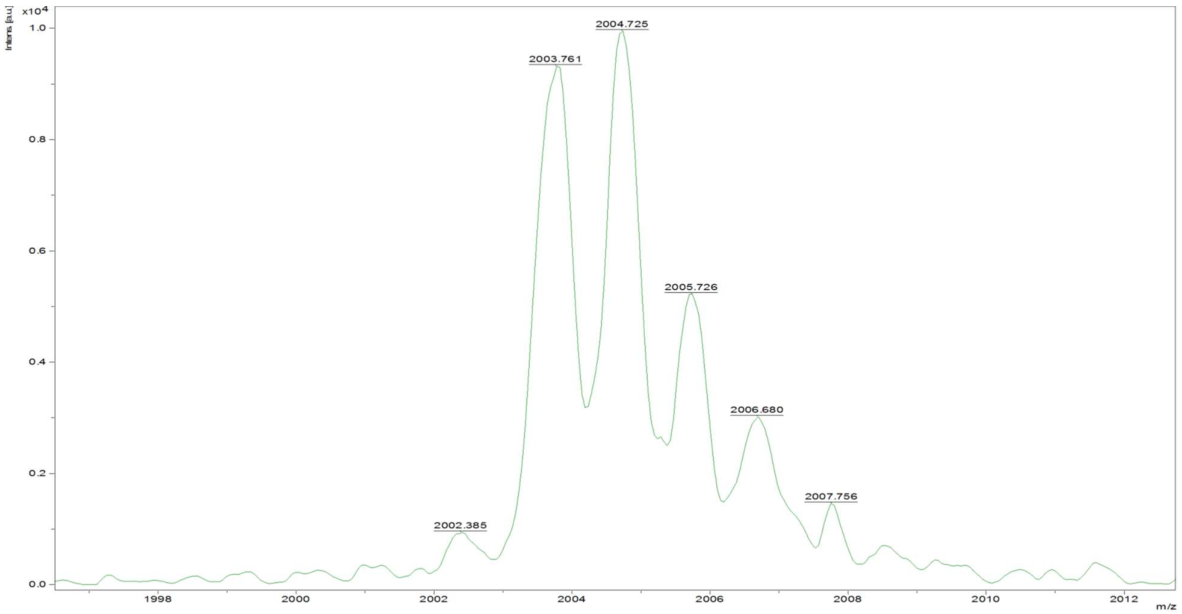

G₃Per = 5'-TpTpGpGpGp**X**-3'

X =

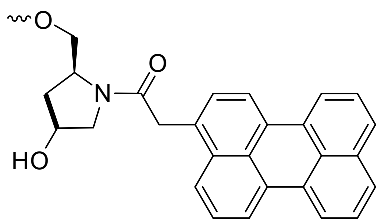

RP-HPLC profile

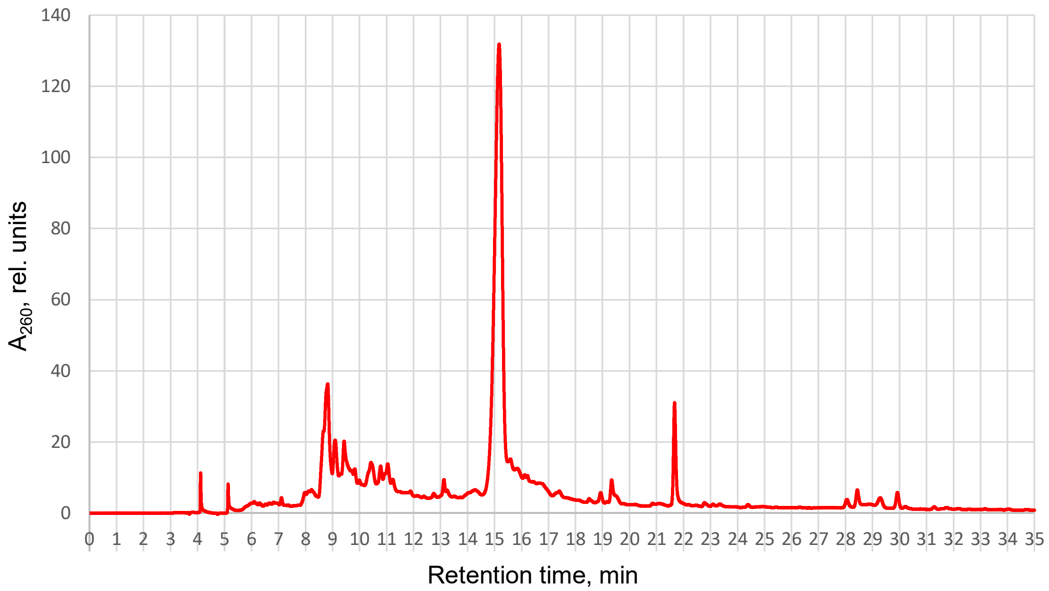

MALDI-TOF MS

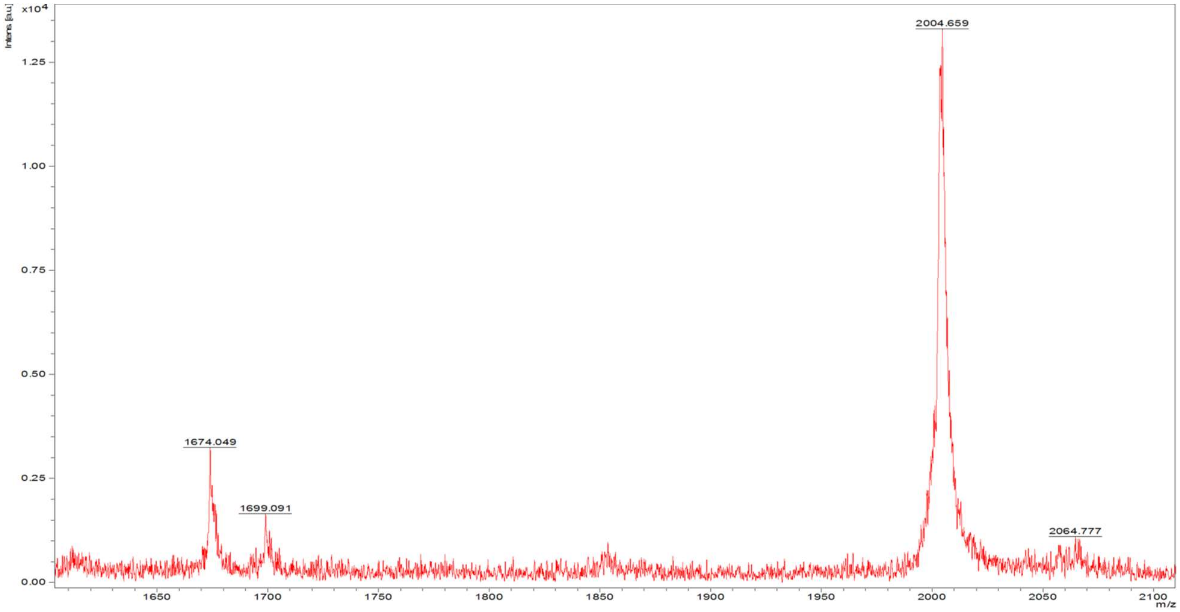

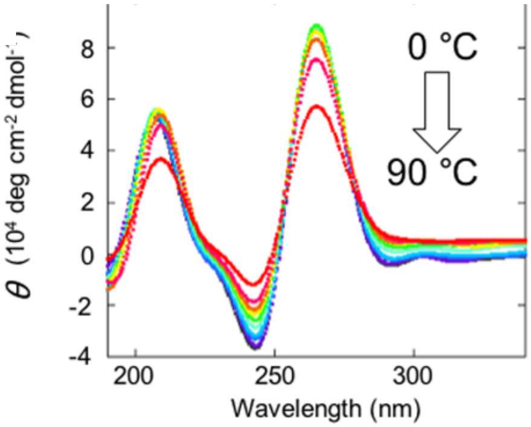

CD spectra of 10 μM VEGF native G4. All the experiments were performed in a buffer consisting of 10 mM Tris- HCl (pH 7.5), 8 mM $MgCl_2$, and 50 mM KCl [1].

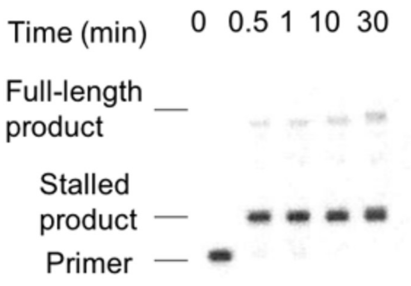

Denaturing-PAGE images of replication products from a native VEGF containing template in a buffer consisting of 10 mM Tris-HCl (pH 7.5), 8 mM $MgCl_2$, and 10 mM KCl with 1 μM primer, 1 μM template, 250 μM dNTPs, and 1 μM KF exo- at 37 °C [1].

Reference:

[1] Takahashi, S.; Kim, K. T.; Podbevsek, P.; Plavec, J.; Kim, B. H.; Sugimoto, N., Recovery of the Formation and Function of Oxidized G-Quadruplexes by a Pyrene-Modified Guanine Tract. *J. Am. Chem. Soc.* **2018,** 140, (17), 5774-5783.
